# Strategies for COVID-19 Epidemiological Surveillance in India: Overall Policies Till June 2021

**DOI:** 10.3389/fpubh.2021.708224

**Published:** 2021-07-22

**Authors:** Nimisha Ghosh, Indrajit Saha, Jnanendra Prasad Sarkar, Ujjwal Maulik

**Affiliations:** ^1^Department of Computer Science and Information Technology, Institute of Technical Education and Research, Siksha ‘O' Anusandhan (Deemed to be University), Bhubaneswar, India; ^2^Department of Computer Science and Engineering, National Institute of Technical Teachers' Training and Research, Kolkata, India; ^3^Larsen & Toubro Infotech Ltd., Pune, India; ^4^Department of Computer Science and Engineering, Jadavpur University, Kolkata, India

**Keywords:** coronavirus, COVID-19, lockdown, pandemic, SARS-CoV-2, unlock, partial lockdown

## Abstract

Coronavirus disease 2019 (COVID-19), caused by severe acute respiratory syndrome coronavirus 2 (SARS-CoV-2), has gripped the entire world, almost paralysing the human race in its entirety. The virus rapidly transmits *via* human-to-human medium resulting in a massive increase of patients with COVID-19. In order to curb the spread of the disease, an immediate action of complete lockdown was implemented across the globe. India with a population of over 1.3 billion was not an exception and took the challenge to execute phase-wise lockdown, unlock and partial lockdown activities. In this study, we intend to summarise these different phases that the Government of India (GoI) imposed to fight against SARS-CoV-2 so that it can act as a reference guideline to help controlling future waves of COVID-19 and similar pandemic situations in India.

## 1. Introduction

**Figure 1:**
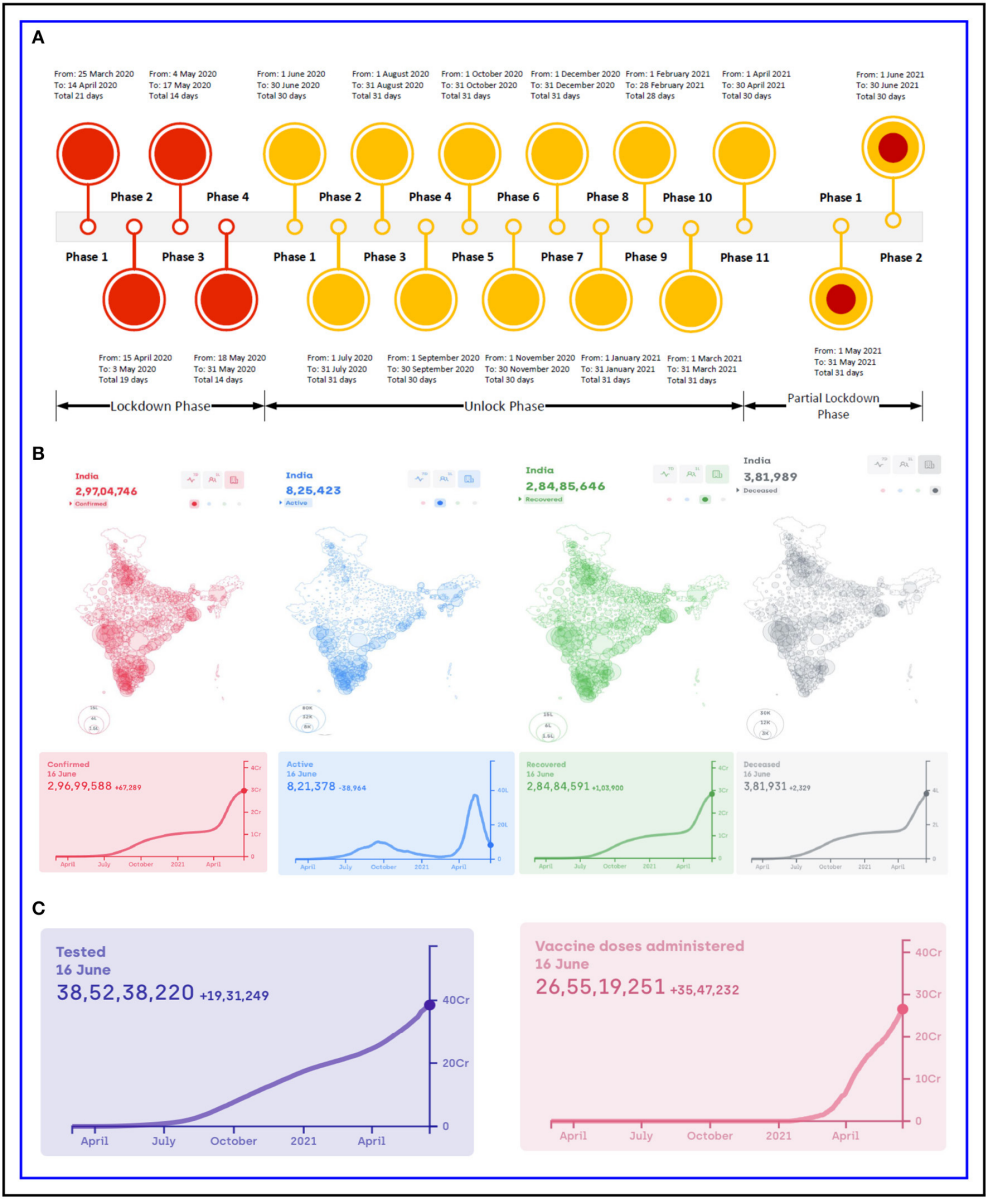
India: **(A)** Chronology of lockdown, unlock phases and partial lockdown phases, **(B)** number of confirmed, active, recovered and deceased cases and **(C)** total number of people tested and vaccine doses administered.

Till now, coronavirus disease 2019 (COVID-19), the disease caused by severe acute respiratory syndrome coronavirus 2 (SARS-CoV-2), has resulted in more than 177 million people being affected and over 3 million deaths as reported in ‘worldometers.info'^1^. In the initial phases of the pandemic, neither any vaccine nor any therapeutic drugs were at the disposal of the human population, thereby making prevention the only way to curb the spread of the virus. Despite active research studies (1–7) in developing preventive vaccine which started quite soon, immediate action was necessary to stop the rapid spread of the virus *via* human-to-human transmission (8, 9), thereby making lockdown the only viable option around the globe; India being no exception. However, imposing lockdown in India, which has a population of over 1.3 billion, is no mean task. Due to the various challenges of implementing lockdown in India, Government of India (GoI) adopted a strategy for COVID-19 epidemiological surveillance by imposing phase-wise lockdown and unlock, which has been found to be a standout example to curb the outbreak. There are studies that have shown the effect of strategies to control the COVID-19 pandemic in the dental field (10), in hospital (11) and in long-term care facilities (LTCFs) (12), by reducing social mixing (13), pertaining to the laboratory-confirmed interval (14) and diagnosis and treatment of patients with cancer (15) during the pandemic. Furthermore, pharmacological (16) and behavioural strategies (17) for the prevention of COVID-19 and the prevention of the same through contact tracing, screening, quarantine, and isolation (18) have also been studied in several research studies. However, there is a distinct lack of study that shows the steps taken by the GoI to prevent the spread of COVID-19 in India. To bridge this gap, in this study we have reported the steps the GoI has taken in different lockdown and unlock phases and finally summarised our view in order to face battle against a similar future pandemic. Figure 1A shows a chronology of the lockdown, unlock and partial lockdown periods of India based on the data collected from the portal of the Ministry of Home Affairs (MHA)^2^, Figure 1B shows the confirmed, active, recovered and death cases in India as of 16th June 2021 and also the trend of the respective cases for the past 1 year, and Figure 1C reports the number of people tested and doses of vaccine administered in the country. It is to be noted that Figures 1B,C are collected from https://www.covid19india.org/. India is currently the second most affected country in the world. It is worth mentioning that the current surge in the pandemic situation of India can be attributed to different circulating variants, especially B.1.617, which is currently the dominant strain in the country and has been deemed as a ‘variant of concern' by WHO (19), and the one of most concern being B.1.617.2, first detected in India in December 2020. B.1.617.2, also known as Delta variant has also recently been classified as ‘variant of concern'^3^. Apart from B.1.617, other variants contributing to this pandemic are B.1.1.7 and B.1.618 first identified in the United Kingdom and West Bengal, an Indian state, respectively. The new variants are also reported to be related to reinfections, either after being afflicted with COVID-19 or after vaccination (20).

## 2. Lockdown, Unlocking and Partial Lockdown Strategies

### 2.1. Strategic Lockdown Phases

The GoI announced a 14-h public curfew on 22nd March 2020, and subsequently, nationwide phase1 complete lockdown was announced on 25th March for 21 days to enforce social distancing with regularly modified guidelines of lockdown measures. When active cases in India crossed the 10,000 mark, phase 2 lockdown was imposed on 15th April for 19 days. After a comprehensive review, the MHA extended the lockdown period for further 2 weeks effective from 4th May (lockdown phase 3). In this period, based on risk profiling, different geographical locations (called districts) were categorised into red (hotspot), green and orange zones, where the green and orange zones were permitted certain relaxations. The districts were marked red zones based on doubling rate of confirmed cases and green zones with the criteria of zero confirmed cases till date or no confirmed cases in the last 21 days. Other districts that neither fall in the red nor in the green zones were considered as orange zone. Moreover, the most sensitive areas of the country were designated as the containment zones. In this time, the National Informatics Centre under the Ministry of Electronics and Information Technology (MeitY) developed a mobile app, Arogya Setu, to identify corona clusters in the country and received appreciation from the WHO as well. The lockdown phase 4 started on 18th May for 14 days. Except at containment zone, it allowed certain activities like sports and inter state travel only for specific purposes. All the declared lockdown-related orders by the MHA, GoI, are reported in Supplementary Table S1.

### 2.2. Strategic Unlock Phases

Total 11 phases of unlock have been executed in the country since 1st June 2020 till now. However, based on the severity of the local situation, individual states were allowed to decide the continuation of lockdown in containment zones. Table 1 reports the summary of guidelines of each unlock phase for important activities. From 8th June onwards, certain public activities were allowed but under strict guidelines. After assessment of the new infections and death cases, unlock phase 2 started on 1st July for a month with the calibrated expansion of domestic travel, while night curfew was active between 10 p.m. and 5 a.m. Barring night curfew, all the restrictions of phase 2 were continued in unlock phase 3, which started on 1st August. The educational institutions were advised to continue online learning. Similarly, public gathering was also prohibited. Only few international travels were allowed by adhering to Standard Operating Procedure the standard operating procedure (SOP). The containment zones were still under strict perimeter control. On 1st September, unlock phase 4 eased additional restrictions outside the containment zones. Students of classes 9–12 and research scholars (Ph.D.) were permitted to visit schools/colleges in case of urgency, whereas 50% of teaching and non-teaching staff were allowed for online teaching. Similarly, in a graded manner from 7th September, training institutes, metro railways and from 21st September certain public gatherings with a ceiling of 100 people were permitted. Unlock phase 5 started on 1st October, while on 15th October new SOPs issued by the Ministry of Youth Affairs and Sports (MoYA&S), the Ministry of Information and Broadcasting, the Ministry of Health and Family Welfare (MoHFW) and the Department of Commerce allowed additional operations like sports training grounds, cinemas/theatres/multiplexes with 50% capacity, entertainment parks, business-to-business (B2B) exhibitions outside the containment zones, intra- and inter-state movements. The guidelines were kept unchanged for unlock phase 6 that started in November and phase 7 that started in December. As an effect of strategic lockdown and unlock so far, the number of active COVID-19 cases declined to 4.5 lakhs as opposed to 10 lakhs on 18th September. However, some places experienced a rise in COVID-19 cases due to the festival season, thus leading to continuation of further unlock phase 8 on 1st January 2021 and phase 9 on 1st February 2021 until further notice. Unlock phase 10 announced on 1st March 2021 followed the same regulations as the earlier phases. Although, several laudable measures taken by the GoI was able to curb the growth of the active cases for 5 months, from the middle of March, there has been a sharp spike in the number of cases as is evident from Figure 1B. This was considered to be the beginning of the second wave of COVID-19 in India. On 11th April 2021 alone, 169,000 people were affected with COVID-19. In the wake of this sudden surge, unlock phase 11 was announced on 23rd March that remained effective till 30th April. It has the same protocols as announced earlier, but additionally test-track-treat protocol also needed to be strictly followed. This protocol included aggressive testing to detect early cases of COVID-19, timely isolation of positive cases, contact tracing and also demarcation of containment zones. Also, the reverse transcription quantitative real-time PCR (RT-qPCR) test was instructed to increase to the prescribed level of 70% or more. Several measures were also introduced by the Ministry of Personnel, Public Grievances and Pensions, Department of Personnel & Training (DoPT) to contain the spread of COVID-19 among central government officials as well. Additionally, the GoI also launched vaccine of AstraZeneca Covishield^4^, manufactured by the Serum Institute of India and Covaxin vaccine^5^ manufactured by the Bharat Biotech Pharma Company with effect from 16th January 2021^6^. By 11th April 2021, over 100 million people in India have been fully or partially vaccinated, while 11 million have been fully vaccinated. All the declared unlock-related orders by the MHA and DoPT, GoI, are reported in Supplementary Table S1.

**Table 1:** Summary of guidelines for unlock and partial lockdown phases.

	**Phase 1**	**Phase 2**	**Phase 3**	**Phase 4**	**Phase 5**	**Phase 6**	**Phase 7**	**Phase 8**	**Phase 9**	**Phase 10**	**Phase 11**	**Phase 1 (Partial Lockdown)**	**Phase 2 (Partial Lockdown)**
Containment Zones	Restriction	Restriction	Restriction	Restriction	Restriction	Restriction	Restriction	Restriction	Restriction	Restriction	Restriction	Restriction	Restriction
Night curfew	9 p.m.–5 a.m.	10 p.m.–5 a.m.	No	No	No	No	Need basis	Need basis	Need basis	Need basis	Need basis	Need basis	Need basis
Use of Aarogya Setu App	Must	Must	Must	Must	Must	Must	Must	Must	Must	Must	Must	Must	Must
Face cover at outside	Must	Must	Must	Must	Must	Must	Must	Must	Must	Must	Must	Must	Must
Social distance	6 feet must	6 feet must	6 feet must	6 feet must	6 feet must	6 feet must	6 feet must	6 feet must	6 feet must	6 feet must	6 feet must	6 feet must	6 feet must
Legal action of violating guideline	Applicable	Applicable	Applicable	Applicable	Applicable	Applicable	Applicable	Applicable	Applicable	Applicable	Applicable	Applicable	Applicable
Spitting in public places	Punishable	Punishable	Punishable	Punishable	Punishable	Punishable	Punishable	Punishable	Punishable	Punishable	Punishable	Punishable	Punishable
Person ≥ 65 years old	Home stay	Home stay	Home stay	Home stay	Home stay	Home stay	Home stay	Home stay	Home stay	Home stay	Home stay	Home stay	Home stay
Person with co-morbidities	Home stay	Home stay	Home stay	Home stay	Home stay	Home stay	Home stay	Home stay	Home stay	Home stay	Home stay	Home stay	Home stay
Pregnant women	Home stay	Home stay	Home stay	Home stay	Home stay	Home stay	Home stay	Home stay	Home stay	Home stay	Home stay	Home stay	Home stay
Children ≤ 10 years old	Home stay	Home stay	Home stay	Home stay	Home stay	Home stay	Home stay	Home stay	Home stay	Home stay	Home stay	Home stay	Home stay
Religious places	Allowed	Allowed	Allowed	Allowed	Allowed	Allowed	Allowed	Allowed	Allowed	Allowed	Allowed	Closed	Closed
Hotels	Allowed	Allowed	Allowed	Allowed	Allowed	Allowed	Allowed	Allowed	Allowed	Allowed	Allowed	Allowed	Allowed
Restaurants	Allowed	Allowed	Allowed	Allowed	Allowed	Allowed	Allowed	Allowed	Allowed	Allowed	Allowed	Only home delivery	Only home delivery
Bars	Prohibited	Prohibited	Prohibited	Prohibited	Prohibited	Prohibited	Prohibited	Prohibited	Prohibited	Prohibited	Prohibited	Prohibited	Prohibited
Other hospitality services	Allowed	Allowed	Allowed	Allowed	Allowed	Allowed	Allowed	Allowed	Allowed	Allowed	Allowed	Allowed	Allowed
Shopping Malls	Allowed	Allowed	Allowed	Allowed	Allowed	Allowed	Allowed	Allowed	Allowed	Allowed	Allowed	Closed	Closed
Number of customer at a time at shop	≤ 5	≤ 5	≤ 5	≤ 5	≤ 5	≤ 5	≤ 5	≤ 5	≤ 5	≤ 5	≤ 5	≤ 5	≤ 5
Physical distance among customers	6 feet must	6 feet must	6 feet must	6 feet must	6 feet must	6 feet must	6 feet must	6 feet must	6 feet must	6 feet must	6 feet must	6 feet must	6 feet must
Educational institutes	Closed	Closed	Closed	Partial	Partial	Partial	Partial	Partial	Partial	Partial	Partial	Partial	Partial
Online/distance learning	Encouraged	Encouraged	Encouraged	Encouraged	Encouraged	Encouraged	Encouraged	Encouraged	Encouraged	Encouraged	Encouraged	Encouraged	Encouraged
Work from home	Encouraged	Encouraged	Encouraged	Encouraged	Encouraged	Encouraged	Encouraged	Encouraged	Encouraged	Encouraged	Encouraged	Encouraged	Encouraged
Train travel	Closed	Selective permit	Selective permit	Selective permit	Selective permit	Selective permit	Allowed	Allowed	Allowed	Allowed	Allowed	Allowed	Allowed
International air-travel	Closed	Selective permit	Selective permit	Selective permit	Selective permit	Selective permit	Selective permit	Selective permit	Selective permit	Selective permit	Selective permit	Selective permit	Selective permit
Metro Railways	Closed	Closed	Closed	Allowed	Allowed	Allowed	Allowed	Allowed	Allowed	Allowed	Allowed	Allowed	Allowed
Cinema Halls	Closed	Closed	Closed	Closed	with 50% capacity	with 50% capacity	with 50% capacity	with 50% capacity	with 50% capacity	with 50% capacity	with 50% capacity	Closed	Closed
Entertainment Parks	Closed	Closed	Closed	Closed	Allowed	Allowed	Allowed	Allowed	Allowed	Allowed	Allowed	Closed	Closed
Closed room Theatres	Closed	Closed	Closed	Closed	with 50% capacity	with 50% capacity	with 50% capacity	with 50% capacity	with 50% capacity	with 50% capacity	with 50% capacity	Closed	Closed
Open Air Theatres	Closed	Closed	Closed	Allowed	Allowed	Allowed	Allowed	Allowed	Allowed	Allowed	Allowed	Closed	Closed
Exhibition Hall	Closed	Closed	Closed	Allowed for B2B	Allowed for B2B	Allowed for B2B	Allowed for B2B	Allowed for B2B	Allowed for B2B	Allowed for B2B	Allowed for B2B	Allowed for B2B	Allowed for B2B
Auditoriums	Closed	Closed	Closed	Closed	≤ 200 people	≤ 200 people	≤ 200 people	≤ 200 people	≤ 200 people	≤ 200 people	≤ 200 people	Closed	Closed
Academic functions	Prohibited	Prohibited	Prohibited	≤ 100 people	≤ 100 people	≤ 100 people	≤ 200 people	≤ 200 people	≤ 200 people	≤ 200 people	≤ 200 people	Prohibited	Prohibited
Religious gathering	Prohibited	Prohibited	Prohibited	≤ 100 people	≤ 100 people	≤ 100 people	≤ 200 people	≤ 200 people	≤ 200 people	≤ 200 people	≤ 200 people	Prohibited	Prohibited
Social gathering	Prohibited	Prohibited	Prohibited	≤ 100 people	≤ 100 people	≤ 100 people	≤ 200 people	≤ 200 people	≤ 200 people	≤ 200 people	≤ 200 people	Prohibited	Prohibited
Political gathering	Prohibited	Prohibited	Prohibited	≤ 100 people	≤ 100 people	≤ 100 people	≤ 200 people	≤ 200 people	≤ 200 people	≤ 200 people	≤ 200 people	Prohibited	Prohibited
Cultural gathering	Prohibited	Prohibited	Prohibited	≤ 100 people	≤ 100 people	≤ 100 people	≤ 200 people	≤ 200 people	≤ 200 people	≤ 200 people	≤ 200 people	Prohibited	Prohibited
Marriage gathering (permitted number of guests)	≤ 50	≤ 50	≤ 50	≤ 50	≤ 50	≤ 50	≤ 50	≤ 50	≤ 50	≤ 50	≤ 50	≤ 50	≤ 50
Funeral gathering (permitted number of people)	≤ 20	≤ 20	≤ 20	≤ 20	≤ 20	≤ 20	≤ 20	≤ 20	≤ 20	≤ 20	≤ 20	≤ 20	≤ 20
Entertainment functions	Prohibited	Prohibited	Prohibited	≤ 100 people	≤ 100 people	≤ 100 people	≤ 200 people	≤ 200 people	≤ 200 people	≤ 200 people	≤ 200 people	Prohibited	Prohibited
Other large congregations	Prohibited	Prohibited	Prohibited	≤ 100 people	≤ 100 people	≤ 100 people	≤ 200 people	≤ 200 people	≤ 200 people	≤ 200 people	≤ 200 people	Prohibited	Prohibited
Sports	Closed	Closed	Closed	≤ 100 people	≤ 100 people	≤ 100 people	≤ 200 people	≤ 200 people	≤ 200 people	≤ 200 people	≤ 200 people	Closed	Closed
Gymnasiums	Closed	Closed	Closed	≤ 100 people	≤ 100 people	≤ 100 people	≤ 200 people	≤ 200 people	≤ 200 people	≤ 200 people	≤ 200 people	Closed	Closed
Yoga Institutes	Closed	Closed	Closed	≤ 100 people	≤ 100 people	≤ 100 people	≤ 200 people	≤ 200 people	≤ 200 people	≤ 200 people	≤ 200 people	Closed	Closed
Swimming Pools	Closed	Closed	Closed	Closed	Partial	Partial	Partial	Partial	Partial	Partial	Partial	Closed	Closed
Vaccine implementation	-	-	-	-	-	-	-	Started	Continuing	Continuing	Continuing	Continuing	Continuing

The surveillance response to COVID-19 by the GoI since March 2020 can be summarised into the following primary strategic steps:

- Impose complete lockdown and strategic unlocks on the entire population with well-defined SOP for shorter period minimising socio-economic impact.
- Assess the impact and severity in daily life as diverse cultures have different priorities.
- Based on a comprehensive review, individual state of the country needs a native SOP for regulating public activities.
- Such regulation needs to be followed for certain periods with strict regular assessment until daily new infection and death cases are under control before starting restriction unlocking.
- In unlock phases, based on the risk profiling, divide the inter-geographical locations into different categories and mark them according to probable severity. While restrictions can be relaxed at low-risk zone, strict surveillance is required in a high-risk zone.
- Until the entire population is found safe, such regulated unlock phases and if necessary partial lockdown phases need to be followed with strictly defined guidelines.
- At the same time, age- and risk-wise vaccination plan needs to be prepared if the vaccine is available.

### 2.3. Partial Lockdown Phases

Despite the aforementioned stringent protocols, India started facing the worst surge of the pandemic from the mid of April 2021 onwards. As per the official record on 10th May 2021, over 388,000 people were afflicted. In this situation, partial lockdown phase1 was announced with renewed guidelines and lockdown-like measures for 14 days that later extended till the end of the month. The measures included renewal of night curfew, prohibiting social, political, entertainment, academic, cultural, religious and festival-related gatherings and congregations, closing of shopping complexes, cinema halls, restaurants and bars, sports complexes, gym, spas, swimming pool and religious places, while essential services were allowed to operate as usual. Apart from these, public transport such as railways, metros, buses and cabs were instructed to operate at a maximum capacity of 50%. DoPT also introduced new measures on 6th May 2021 to curb the spread of COVID-19 among government officials. Based on the situation, local governments of states/union territories (UTs) were instructed to take further decisions. Considering COVID-19 afflicted government officials, DoPT had also modified leave regulations in order to help them out. The decision of containment zone for each state/UT as based on the broad-based framework that encompasses more than 10% of people tested positive in a week or bed occupancy that is more than 60% on either oxygen-supported or intensive care unit (ICU) beds. The vaccination programme was encouraged as usual. In fact from 1st May 2021, the government opened vaccination drive for everyone aged 18 and above. On 1st May, it was announced by the GoI that 25% of all procurement for the vaccination programme would be carried out by state governments, while in the mid-June, the GoI reverted to the old system where the Centre can procure 75% of the doses of vaccines and will provide them free of cost to the state governments. Though the partial lockdown phase 1 showed results in the form of declining cases, the number of active and death cases being still very high, the restrictions of partial lockdown phase 1 continued with phase 2 that will now go on till 30th June. All the declared partial lockdown-related orders by the MHA and the DoPT, GoI, are reported in Supplementary Table S1.

**Figure 2:**
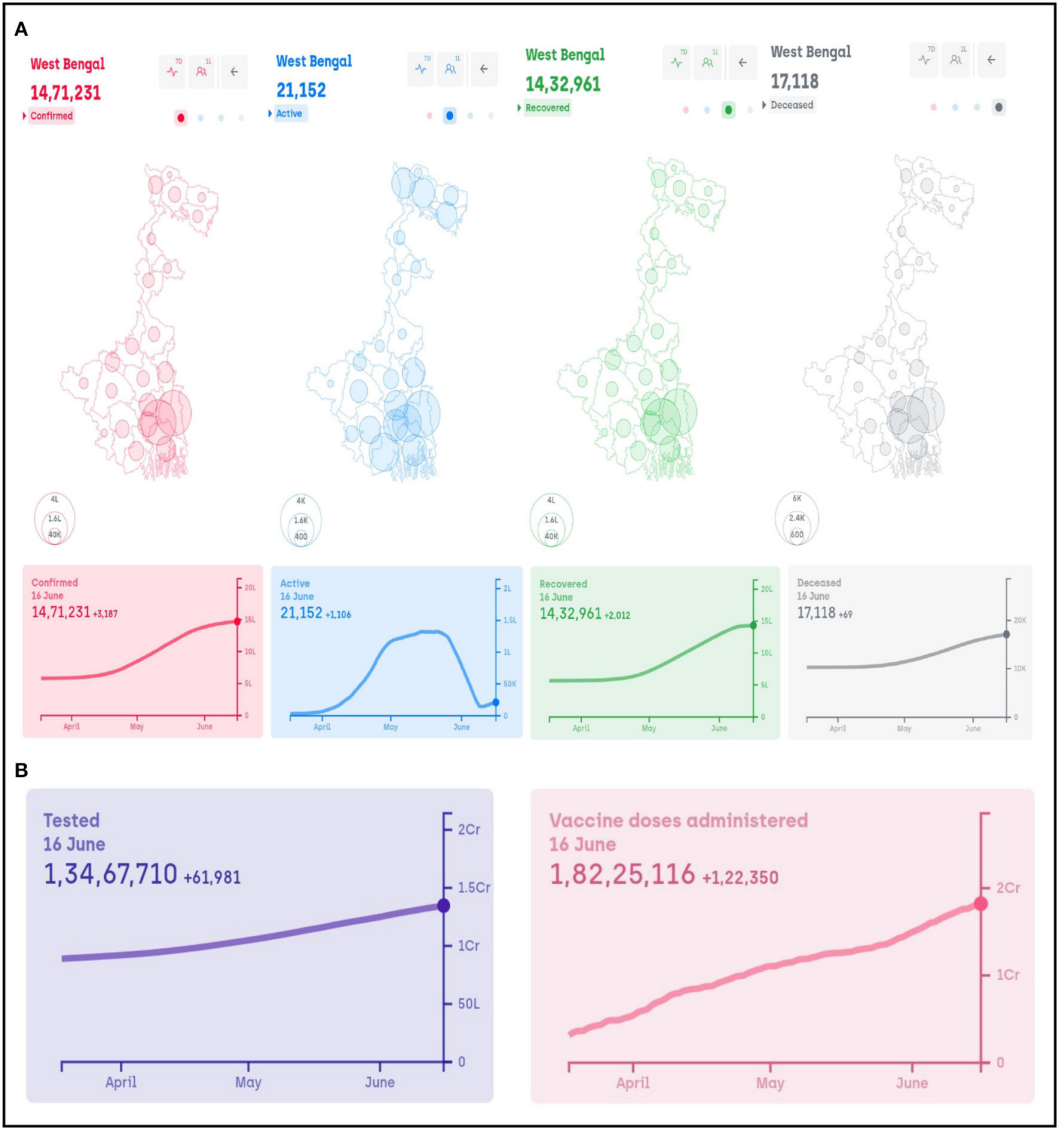
West Bengal: **(A)** number of confirmed, active, recovered and deceased cases and **(B)** total number of people tested and vaccine doses administered.

As mentioned earlier, each state can also independently take additional lockdown measures and these may vary from state to state as well. For example, on 5th May when the number of confirmed cases in the state was 17,583 the Government of West Bengal declared lockdown-like measures. Stand-alone shops were allowed to remain open from 7 a.m. to 10 a.m. and 5 p.m. to 7 p.m., whereas jewellery shops were allowed from 12 p.m. to 3 p.m. on weekdays. Tea gardens and other industries were allowed to operate with 50% staff. Marriage functions were allowed with 50 people, while funeral services were restricted to 20 people. On 15th May when the number of cases was 20,085, the Government of West Bengal declared a further lockdown-like measures from 6 a.m. of 16th May to 6 p.m. of 30th May, which included the opening of retail shops and supplies, vegetables, fruits and grocery shops to operate from 7 a.m. to 10 a.m. The sweetmeat shops were allowed to remain open between 10 a.m. and 5 p.m., whereas for jewellery and saree shops the timing was between 12 p.m. and 3 p.m. All intra-state local trains, metro services, intra-state bus services and inland water transport were instructed to remain closed except for emergency services. Operations in tea gardens and jute mills were allowed with 50 and 30% capacity, respectively, in each shift. All outdoor activities and movement of vehicles were prohibited from 9 p.m. to 5 a.m. except for emergency situations. These restrictions were carried forward till 15th June. Following these measures, the number of cases in West Bengal came down to 4,371 on 15th June. In the light of this, new regulations were put forth by the Government of West Bengal from 15th June that will remain in effect till 30th June. In this regard, parks are allowed to remain open for morning walks and physical exercises from 6 a.m. to 9 a.m. for vaccinated people only. Shops that were allowed to operate between 7 a.m. and 10 a.m. are now allowed till 11 a.m. All the other shops may remain open from 11am to 6pm. Restaurants and bars may remain open from 12 p.m. to 8 p.m. with a 50% seating capacity. Retail shops in shopping malls and market complexes are allowed to remain open during 11 a.m.–6 p.m. with 25% workforce and 30% customers. Games and sports are allowed to resume albeit without any spectators. Indoor/ outdoor shooting and associated activities related to TV programmes and cinemas may resume with maximum of 50 persons per unit at a time, while IT sectors are allowed to function with 50% capacity in each shift subjected to following of all COVID-related norms. Private and corporate offices are also allowed to operate from 10 a.m. to 4 p.m. with a maximum of 25% of total strength. Figure 2A shows the confirmed, active, recovered and death cases in West Bengal as of 16th June and (Figure 2B) reports the number of people tested and doses of vaccine administered in the state. All these figures are considered from https://www.covid19india.org/. Three lockdown-related orders dated 15th May onwards by the Government of West Bengal are provided in Supplementary Table S1.

## 3. Economy and Health During the Pandemic

With complete lockdown during the first wave and intermittent lockdowns during the second one, Indian economy has been devastated, to say the least. The fall in gross domestic product (GDP) in April-June quarter of 2020 was at a record low of 23.9%. The economy somewhat revived in the second quarter with a growth of (-) 7.5%. The poor population of the country are affected the most. In this scenario, the measures aimed at the poor population of the country are as follows^7^:

- Free food grain for 80 crore (800 million) people, free cooking gas for 8 crore (80 million) families and direct cash transfer to over 40 crore (400 million) farmers, women, elderlies, the poor and the needy that were provided under Pradhan Mantri Garib Kalyan (Prime Minister Scheme for Upliftment of Poor People) Package, which is valued at Rs. 2.76 lakh crore (2,760 billion).
- With an expenditure of Rs. 39,293 crore (392.93 billion) under Pradhan Mantri Garib Kalyan Rojgar Abhiyan (Prime Minister Scheme for Employment of Poor People), 50.78 crore (507.8 million) person-days of employment were generated.
- Rs. 30,000 crores (300 billion) Additional Emergency Working Capital Funding for farmers through National Bank for Agriculture and Rural Development (NABARD) is being provided. Rs. 25,000 crores (250 billion) has been disbursed so far as on 4th December 2020.

Structural reforms were also announced as part of the AtmaNirbhar Bharat (Self-reliant India) Package to support micro-enetrprise, small and medium enterprises. Prime Minister Mr. Narendra Modi has also announced free ration for poor under Prime Minister Garib Kalyan Yojana till November 2021. Apart from these, union budget 2021-22 has also announced measures to foster the economic development listed under health and wellbeing, physical and financial capital and infrastructure, inclusive development for aspirational India, reinvigorating human capital, innovation and research and development (R&D) and minimum government and maximum governance. At a recent ViVaTech summit, as a post-pandemic development, the GoI has resolved to focus on repair and prepare. Although there has been a huge impact on the economy, India has implemented huge reforms in mining, space, banking, atomic energy, etc.^8^. Moreover, the individual state governments have come up with various schemes to resurrect the economy and help the needy.

Another sector that has been directly hit by this ongoing pandemic is healthcare. The current pandemic has put a huge stress on the already restricted healthcare system of India. With a very meagre number of government doctors [1 for every 10,926 persons (21)], the need to cater to the huge population has been difficult to meet. Currently, in the second wave of the pandemic, India has seen an acute shortage of oxygen supply and basic medicines. There is very little or no infrastructure in the government hospitals to meet the demands of the people who have been suffering from COVID-19. The doctors have been so overwhelmed with their current duty that patients with non-COVID emergencies could not be properly treated. Recently, to mitigate the shortage of oxygen, the GoI has launched ‘Project O2 for India'. On top of the crisis that India is already facing due to COVID-19, post-COVID fungal disease like mucormycosis has gripped the population. Although the second wave is on the wane now, the GoI should seriously consider reviving its policies and prepare itself with better strategies if and when third wave hits the country.

## 4. Averting Any More Waves of COVID-19

One of the main reasons for this surge has been attributed to the lax behaviour of people towards following COVID-appropriate norms. However, even though the government has taken various measures, the government cannot be spared as well. Although the fight against the virus started on a very positive and stringent note in March 2020, once the number of cases started dropping, the COVID-19 taskforce created by the government took the matter lightly (22, 23). There has been lacuna on the part of the government that cannot be ignored wherein religious and mass gatherings were allowed at rampant. All these cumulatively led to the unfortunate second wave in the country. Also, the vaccination drive is on the wane as well. While 100 million people got vaccinated by 11th April, data on 10th May showed only 170 million have been vaccinated. It is high time that the federal government in conjunction with the state governments should take strict measures and ramp up the vaccination drive to mitigate the tumultuous situation the country is facing right now. Although the second wave is subsiding, it has left the country devastated in its wake. With the looming third wave in the near future, we are looking forward that in the coming months the government will take revised measures to increase the vaccination drive, make the public understand the necessity of masking, social distancing, halting mass gatherings, voluntary quarantine and testing and if necessary even a federal lockdown in order to control or even avert the third wave. In fact, quarantine has been proven to avert 44–81% of incident cases and 31–63% of deaths (18). With respect to the vaccination strategy, India can follow the example of Chile for vaccine import where Chile did not disregard the Chinese vaccine Sinovac even for political reasons (24). In this pandemic situation, vaccines are our biggest weapons. Still, the vaccination situation is very grim in India with only 3.7% and 16.2% of the population being fully and partially vaccinated, respectively. Studies conducted by Rossman et al. (25) and Leshem and Wilder-Smith (26) show the importance of vaccination. In the study of Rossman et al. (25), it is reported that there has been a significant decrease in the number of COVID-19 cases and hospitalisation of the vaccinated individuals in Israel. If not anything else, the second wave should teach the GoI the importance of vaccination and to boost the speed of COVID-19 immunisation to curb the spread of COVID-19 and avoid the projected 1.19 million deaths by October 2021. Apart from the suggestions provided in this study,Aiyar et al. (27) and Kuppalli et al. (28) can also be referred for other actions that need to be taken in order to avoid any more waves of COVID-19 in the country.

## 5. Conclusion

The COVID-19 pandemic has been a wakeup call for everyone that has led to many to make significant changes in their respective life. Also, the pandemic has given us a learning opportunity to battle against similar pandemics in the future. Though vaccination drive has already begun in many parts of the world, resurgence of COVID-19 at different times is going on in different parts of the world. Thus, thinking about sustainable long-term strategies is the order of the day. While researchers suspect more pandemics in the future and in case of unavailability of effective drugs, apart from stronger measures like lockdown and vaccination, detailed regulations on daily activities illustrated in this study should be followed as a reference guide to tackle future epidemics and also during the different waves of COVID-19 in the country.

## Author Contributions

NG and IS designed the research. IS, NG, JS, and UM analysed data and wrote the manuscript. All authors reviewed and approved the final version of the manuscript.

## Conflict of Interest

JS is employed by company Larsen & Toubro Infotech Ltd., Pune. The remaining authors declare that the research was conducted in the absence of any commercial or financial relationships that could be construed as a potential conflict of interest.

## Footnotes

^1^https://www.worldometers.info/coronavirus/

^2^https://www.mha.gov.in/

^3^https://www.business-standard.com/article/current-affairs/us-cdc-classifies-delta-variant-of-coronavirus-as-variant-of-concern-121061700267_1.html

^4^https://www.seruminstitute.com/product_covishield.php

^5^https://www.bharatbiotech.com/covaxin.html

^6^https://www.mohfw.gov.in/covid_vaccination/vaccination/index.html

^7^https://pib.gov.in/PressReleasePage.aspx?PRID=1696278

^8^https://www.thehindu.com/news/national/modi-calls-for-repair-and-prepare-as-india-emerges-from-pandemic/article34830793.ece
