# Supplementary material for: Strategies for COVID-19 Epidemiological Surveillance in India: Overall Policies Till June 2021
Source: Front Public Health. 2021 Jul 22;9:708224. doi: 10.3389/fpubh.2021.708224 (PMC8339284; doi:10.3389/fpubh.2021.708224)
Supplement: Supplementary file 1 [file Table_1.pdf]

Supplementary for “Strategies for COVID-19 Epidemiological Surveillance in India: Overall Policies till June 2021”

Nimisha Ghosh[a,e], Indrajit Saha[b,e,*], Jnanendra Prasad Sarkar[c,e], Ujjwal Maulik[d,e]

[a]*Department of Computer Science and Information Technology, Institute of Technical Education and Research, Siksha ‘O’ Anusandhan (Deemed to be University), Bhubaneswar, Orissa, India*
[b]*Department of Computer Science and Engineering, National Institute of Technical Teachers’ Training and Research, Kolkata, West Bengal, India*
[c]*Larsen & Toubro Infotech Ltd, Pune, India*
[d]*Department of Computer Science and Engineering, Kolkata, Jadavpur University, India*
[e]*Equally contributed*

Sl. No.	Ministry / Department (under GoI) Issued Order	Order No.	Phase	Link
1	Ministry of Home Affairs	No. 40-3/2020-DM-I (A) & No. 1-29/2020-PP Dated 24.03.2020	Lockdown	http://www.nitttrkol.ac.in/indrajit/projects/COVID-LockdownUnlock-India/downloads/supplementary/1-Lockdown-24032020.pdf
2	Ministry of Home Affairs	No. 40-3/2020-DM-I (A) & No. 1-137/2018-Mit-II (FTS-10548) Dated 14.04.2020	Lockdown	http://www.nitttrkol.ac.in/indrajit/projects/COVID-LockdownUnlock-India/downloads/supplementary/2-Lockdown-14042020.pdf
3	Ministry of Home Affairs	No. 40-3/2020-DM-I (A) Dated 01.05.2020	Lockdown	http://www.nitttrkol.ac.in/indrajit/projects/COVID-LockdownUnlock-India/downloads/supplementary/3-Lockdown-01052020.pdf
4	Ministry of Home Affairs	No. 40-3/2020-DM-I (A) Dated 17.05.2020	Lockdown	http://www.nitttrkol.ac.in/indrajit/projects/COVID-LockdownUnlock-India/downloads/supplementary/4-Lockdown-17052020.pdf
5	Ministry of Home Affairs	No. 40-3/2020-DM-I (A) Dated 30.05.2020	Unlock	http://www.nitttrkol.ac.in/indrajit/projects/COVID-LockdownUnlock-India/downloads/supplementary/1-Unlock-30052020.pdf
6	Ministry of Home Affairs	No. 40-3/2020-DM-I (A) Dated 29.06.2020	Unlock	http://www.nitttrkol.ac.in/indrajit/projects/COVID-LockdownUnlock-India/downloads/supplementary/2-Unlock-29062020.pdf
7	Ministry of Home Affairs	No. 40-3/2020-DM-I (A) Dated 29.07.2020	Unlock	http://www.nitttrkol.ac.in/indrajit/projects/COVID-LockdownUnlock-India/downloads/supplementary/3-Unlock-29072020.pdf
8	Ministry of Home Affairs	No. 40-3/2020-DM-I (A) Dated 29.08.2020	Unlock	http://www.nitttrkol.ac.in/indrajit/projects/COVID-LockdownUnlock-India/downloads/supplementary/4-Unlock-29082020.pdf
9	Ministry of Home Affairs	No. 40-3/2020-DM-I (A) Dated 30.09.2020	Unlock	http://www.nitttrkol.ac.in/indrajit/projects/COVID-LockdownUnlock-India/downloads/supplementary/5-Unlock-30092020.pdf
10	Ministry of Home Affairs	No. 40-3/2020-DM-I (A) Dated 27.10.2020	Unlock	http://www.nitttrkol.ac.in/indrajit/projects/COVID-LockdownUnlock-India/downloads/supplementary/6-Unlock-27102020.pdf
11	Ministry of Home Affairs	No. 40-3/2020-DM-I (A) Dated 25.11.2020	Unlock	http://www.nitttrkol.ac.in/indrajit/projects/COVID-LockdownUnlock-India/downloads/supplementary/7-Unlock-25112020.pdf
12	Ministry of Home Affairs	No. 40-3/2020-DM-I (A) Dated 28.12.2020	Unlock	http://www.nitttrkol.ac.in/indrajit/projects/COVID-LockdownUnlock-India/downloads/supplementary/8-Unlock-28122020.pdf
13	Ministry of Home Affairs	No. 40-3/2020-DM-I (A) Dated 27.01.2021	Unlock	http://www.nitttrkol.ac.in/indrajit/projects/COVID-LockdownUnlock-India/downloads/supplementary/9-Unlock-27012021.pdf
14	Ministry of Home Affairs	No. 40-3/2020-DM-I (A) Dated 26.02.2021	Unlock	http://www.nitttrkol.ac.in/indrajit/projects/COVID-LockdownUnlock-India/downloads/supplementary/10-Unlock-26022021.pdf
15	Ministry of Home Affairs	No. 40-3/2020-DM-I (A) Dated 23.03.2021	Unlock	http://www.nitttrkol.ac.in/indrajit/projects/COVID-LockdownUnlock-India/downloads/supplementary/11-Unlock-23032021.pdf
	Ministry of Personnel, Public Grievances and Pensions, Department of Personnel & Training	F.No.11013/9/2014-Estt.A-III Dated 19.04.2021		http://www.nitttrkol.ac.in/indrajit/projects/COVID-LockdownUnlock-India/downloads/supplementary/1-DoPT-19042021.pdf
16	Ministry of Home Affairs	No. 40-3/2020-DM-I (A) Dated 29.04.2021	Partial Lockdown	http://www.nitttrkol.ac.in/indrajit/projects/COVID-LockdownUnlock-India/downloads/supplementary/1-PartialLockdown-29042021.pdf
	Government of West Bengal	No. 629-ISS/2M-22/2020 Dated 05.05.2021		http://www.nitttrkol.ac.in/indrajit/projects/COVID-LockdownUnlock-India/downloads/supplementary/1-PartialLockdown-WestBengal-05052021.pdf
	Ministry of Personnel, Public Grievances and Pensions, Department of Personnel & Training	F.No.11013/9/2014-Estt.A-III Dated 06.05.2021		http://www.nitttrkol.ac.in/indrajit/projects/COVID-LockdownUnlock-India/downloads/supplementary/2-DoPT-06052021.pdf
	Government of West Bengal	No. 647-ISS/2M-22/2020 Dated 15.05.2021		http://www.nitttrkol.ac.in/indrajit/projects/COVID-LockdownUnlock-India/downloads/supplementary/2-PartialLockdown-WestBengal-15052021.pdf
17	Ministry of Home Affairs	No. 40-3/2020-DM-I (A) Dated 27.05.2021	Partial Lockdown	http://www.nitttrkol.ac.in/indrajit/projects/COVID-LockdownUnlock-India/downloads/supplementary/2-PartialLockdown-27052021.pdf
	Government of West Bengal	No. 707-ISS/2M-22/2020 Dated 29.05.2021		http://www.nitttrkol.ac.in/indrajit/projects/COVID-LockdownUnlock-India/downloads/supplementary/3-PartialLockdown-WestBengal-29052021.pdf
	Ministry of Personnel, Public Grievances and Pensions, Department of Personnel & Training	No.13020/1/2019-Estt.(L) Dated 07.06.2021		http://www.nitttrkol.ac.in/indrajit/projects/COVID-LockdownUnlock-India/downloads/supplementary/3-DoPT-07062021.pdf
	Government of West Bengal	No. 753-ISS/2M-22/2020 Dated 14.06.2021		http://www.nitttrkol.ac.in/indrajit/projects/COVID-LockdownUnlock-India/downloads/supplementary/4-PartialLockdown-WestBengal-14062021.pdf

Table S1: Link of relevant orders

*Corresponding author: indrajit@nitttrkol.ac.in
